# A New Perspective on the Diagnosis of Septic Arthritis: High-Resolution Thermal Imaging

**DOI:** 10.3390/jcm12041573

**Published:** 2023-02-16

**Authors:** Huseyin Gunay, Ozgur Mert Bakan, Javad Mirzazade, Murat Celal Sozbilen

**Affiliations:** 1 Department of Orthopedics and Traumatology, Ege University, Bornova, 35100 Izmir, Turkey; 2 Department of Orthopedics and Traumatology, Cigli Trainning and Research Hospital, 35100 Izmir, Turkey; 3 Department of Orthopedics and Traumatology, VM Medical Park Hospital, 41140 Kocaeli, Turkey

**Keywords:** septic arthritis, thermal imaging, synovial fluid

## Abstract

Aims: An increase in temperature in an area suspected of septic arthritis is a clinically important finding. The aim of this study is to evaluate temperature changes in septic arthritis with a high-resolution thermal camera. Methods: A total of 49 patients, who were evaluated with a prediagnosis of arthritis (septic or non-septic), were included in this study. A temperature increase in the knee with suspected septic arthritis was evaluated by using thermal imaging and compared with the opposite-side joint. Then, in order to confirm the diagnosis, a culture was taken using routine intra-articular aspiration. Results: The thermal measurements were compared in 15 patients with septic arthritis and 34 patients with non-septic arthritis. The mean temperature was 37.93 °C in the septic group, while it was 36.79 °C in the non-septic group (*p* < 0.000 *). The mean temperature difference in both joints was 3.40 °C in the septic group, while it was 0.94 °C in the non-septic group (*p* < 0.000 *). While the mean temperature was 37.10 °C in the group with septic arthritis, it was measured to be 35.89 °C in the group non-septic arthritis (*p* < 0.020). A very strong positive correlation was found between the difference in the mean temperatures of both groups and the values of the hottest and coldest points (r = 0.960, r = 0.902). Conclusions: In the diagnosis of septic arthritis, thermal imagers can be used as a non-invasive diagnostic tool. A quantitative value can be obtained to indicate to a local temperature increase. In future studies, specially designed thermal devices can be developed for septic arthritis.

## 1. Introduction

Septic arthritis is an acute infective presentation of the joint requiring urgent intervention, and that is why it is difficult to make a differential diagnosis (Matan and Smith 1997) [1].

The differential diagnosis of septic arthritis includes osteomyelitis, juvenile rheumatoid arthritis, and often acute inflammatory arthritis. Hemophilia, malignancy, rheumatoid fever, and various non-rheumatoid autoimmune diseases should also be considered during the course of this procedure. It can be particularly difficult when distinguishing septic arthritis from inflammatory arthritis. In recent studies, this distinction has been emphasized, and diagnostic aid algorithms have been attempted by means of studies containing high evidence value (Kocher et al., 1999, Kocher et al., 2004, Caird et al., 2006) [2,3,4]. 

The importance of the differential diagnosis of septic arthritis and inflammatory arthritis lies in the fact that their treatment modalities vary. While septic arthritis is surgically treated, the treatment of inflammatory arthritis consists of medical and conservative follow-up (Kocher et al., 2003) [5]. The need to determine their differential diagnosis with a rapid and non-invasive method may be groundbreaking during the early treatment and prognosis of these diseases.

An increase in temperature in an area suspected of being present with septic arthritis is one of the clinically important findings. Therefore, this temperature increase can be quantified and viewed through thermal cameras.

The aim and hypothesis of this study is that a thermal camera can be used as a non-invasive auxiliary diagnostic tool in the diagnosis of septic arthritis. Determining the temperature difference in the joint with suspected septic arthritis by using thermal measurement and comparing it with the non-septic joint will help with the diagnosis. With the aid of this device, a quantitative value can be obtained to indicate a local temperature increase in the joint, aside from palpation, which is a remarkable finding in clinical presentation of septic arthritis.

## 2. Patients and Methods

A total of 49 patients, including 15 patients with septic arthritis and 34 patients with non-septic arthritis, were included in this study. Both male and female patient of all ages, who were admitted to an emergency room or were evaluated with a consultation from another clinical unit and who were also present with a pre-diagnosis of arthritis (septic or non-septic) in the knee (with complaints of redness, swelling, pain, effusion, increased temperature, edema, and inability to walk), were included in the study. Patients with extra-articular inflammatory problems, bilateral involvement of the knee, delayed or subacute cases of septic arthritis, and a history of surgery from the same joint were excluded from the study. Local temperature rise was determined by palpation during the examination. Patients who might not have the local temperature rise seen in those with immunosuppressive treatment were excluded. The patients and their parents were informed about the study, and their consents were also obtained.

The demographic data, detailed anamnesis, and physical examination findings of each patient to be included in the study were recorded. Hemogram, C-reactive protein (CRP), sedimentation (sediment), anti-streptolysin O (aso), and biochemistry values, which are routinely requested from patients when they are admitted, were also recorded.

### 2.1. Thermal Imaging (Standardization)

**Figure 1:**
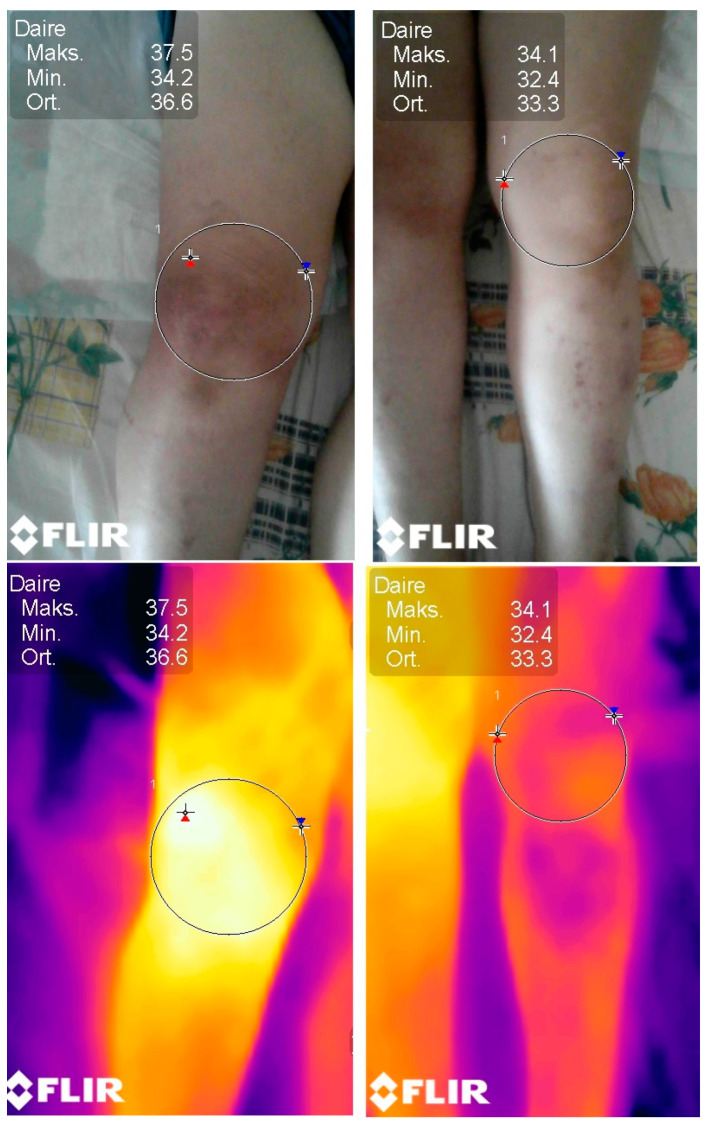
Imaging of a right knee with septic arthritis compared to the contralateral knee using thermal imaging. The difference between the hottest temperature value (red sign), the coldest value (blue sign), and the mean temperature value are comparatively displayed. The mean temperature difference is 3.3 °C.

**Figure 2:**
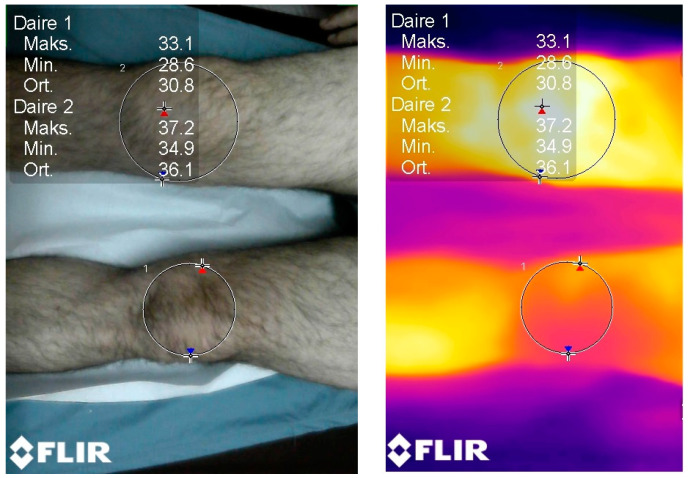
Imaging of a left knee with septic arthritis compared to the contralateral knee using thermal imaging. The difference between the hottest temperature value (red sign), the coldest value (blue sign), and the mean temperature value are comparatively displayed. The mean temperature difference is 1.2 °C.

**Figure 3:**
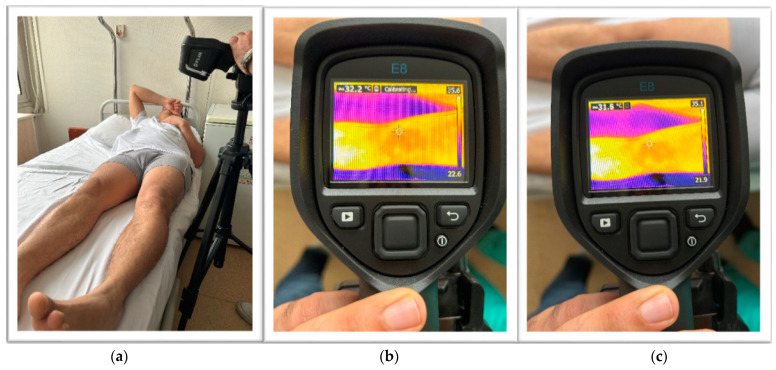
A scenario showing the thermal camera and a patient’s position during imaging (**a**), calibrating (**b**), and shooting (**c**).

After having obtained the optimal room temperature and humidity when the patients were in a supine position, the required measurements were carried out in the same room in the ER. The patients’ malleolus was kept at 10 cm apart from each other, and the camera was stabilized with a tripod at 20 cm above the knees. Upon the initiation of the process, we waited for 3 min before taking the measurements for automatic calibration. Calibration was important and standardized via ISO (simphson rc 2008) [6]. The machine calibration was carried out automatically. As a reference for the measurements, The Glamorgan Protocol reference atlas for clinical thermograph was taken as the reference point (amber k 2008) [7]. Then, thermographic data were obtained with a Flir^®^ E75 model (Flir Systems, Wilsonville, OR, USA) thermal camera, the technical characteristics of which were recorded as the detector type with an uncooled microbolometer of 17 µm; the temperature range was set from −20 °C to 650 °C; the thermal sensitivity was set at 0.03 °C (at 30 °C); the verification of reading accuracy was at ±2 °C or ±2%; the frame rate was 30 Hz; the spectral range (wavelength) was 7.5–14.0 µm; the IR resolution was 320 × 240 (76,800 pixels); the minimum focus distance was 0.5 m; and the weight was 0.850 kg (Figure 1, Figure 2 and Figure 3). 

For a definitive diagnosis of septic arthritis, the routine joint puncture procedure was applied only to the affected joint, while further procedures were carried out by a clinical team, who was knowledgeable about the disease but was independent of the study, within the framework of standard clinical practice. Following these processes, the recorded data were analyzed again using the temperature analytical application (via the software acquired with the camera) to reveal the temperature difference, and the recorded data and laboratory values were compared statistically with the contralateral joint.

Having obtained the first average temperature (average) and the average temperature difference (average difference) values, the hottest spot of the displayed regions was determined. These thermograms were evaluated and compared together. In addition, the relationship between these data and the laboratory data (erythrocyte sedimentation rate, C-reactive protein, etc.) was evaluated.

With the same thermal imaging, the coldest spot (Coldest spot-i: the lowest temperature in the suspected infected joint, and Coldest spot-c: the lowest temperature in the opposite control joint), the hottest spot (Hottest spot-i: the highest temperature in the suspected infected joint, and Hottest spot-c: the highest temperature in the contralateral joint), and the average temperature distribution (Average-i: mean temperature in the suspected infected or symptomatic joint, and Average-c: mean temperature in the contralateral joint), which were obtained from both the affected knee’s and the contralateral knee’s joint temperatures and temperature differences, were compared.

### 2.2. Statistical Analysis

Data analysis was performed using SPSS 21.0 (SPSS Inc., Chicago, IL, USA) and studied at a 95% confidence level. The kurtosis and skewness coefficients were examined to determine the conformity of the measurements to a normal distribution, in which values obtained from the scales that are between +3 and −3 are considered sufficient for normal distribution.

It was seen that the kurtosis and skewness coefficients obtained from the measurement values are between −3 and +3. The *t*-test was used to analyze the differences in the measurements according to the two-group categorical variables. The relationship between the quantitative variables with normality was analyzed using the Pearson correlation test, and the relationship between those without normality was analyzed using the Spearman correlation test. The significance level was set at 0.05.

## 3. Results

The ages of the cases ranged from 1 to 90, and the mean age was 39.89 ± 27.65.

While the rate of those with septic arthritis is found to be 30.6%, the rate of those without septic arthritis is 69.4%. Other categorical variables are also shown in Table 1.

The laboratory values of the cases, the minimum and maximum values, and the average values of the measurements from the thermal camera are shown in Table 2.

The test results for the examination of the measurements in terms of septic arthritis status are presented in Table 3.

We observed a significant difference between the groups with different septic arthritis status in terms of ASO in blood values, sedimentation, and leukocyte count in the joint fluid (*p* < 0.05). (Table 3)

There is also a significant difference between the groups with different septic arthritis status in terms of the Coldest spot-i, the Hottest spot-i, the Hottest spot difference, the Coldest spot difference, the Average-i, and the average difference (*p* < 0.05). (Table 3)

Significant differences can be observed in some values between the pediatric age group and the adult age group in the non-septic arthritis group. There are significant differences between the groups with different ages in terms of the Coldest spot-i, the Hottest spot difference, the Coldest spot difference, and the average difference (*p* < 0.05). In particular, the average temperature difference for those aged 18 and under is 0.45, while the average for those over the age of 18 is 1.25. Accordingly, the mean average difference for those over the age of 18 is significantly higher. The Hottest spot difference average of those aged 18 and under is 0.42, while the average of those over 18 years of age is 1.32. Accordingly, the mean Hottest spot difference for those over 18 is significantly higher (Table 4).

When it comes to those with septic arthritis, there is a significant difference in terms of sedimentation between the groups with different ages (*p* < 0.05).

### 3.1. Relationship between Blood Values and the Hottest Spot-i

The highest temperature value measured in the infected joint is the Hottest spot-i. When the infection values evaluated in the blood are examined, it can be seen that there is a positive moderate relationship between Aso, crp, sedimentation, and the Hottest spot-i, and there is also a positive moderate relationship between these values and the Average-i. There is a moderately positive relationship between the average temperature difference and the Hottest spot-i, as well as a positive moderate relationship between the Hottest spot difference in terms of blood values and the Hottest spot-i (Table 5).

### 3.2. Relationship between Joint Fluid Values and Thermal Measurements

In the joint puncture for a definitive diagnosis, a moderate positive relationship is observed between the leukocyte count in the joint fluid and the Hottest spot difference (r = 0.587), while there is a positive moderate relationship (r = 0.583) with the average difference (Table 5).

### 3.3. The Relationship between the Hottest Spot-i and Other Values

The relationship between the Hottest spot-i, which is the highest temperature measured in the joint with septic arthritis, and other measurements was compared. While there is a moderate positive relationship between the Hottest spot-i and the Hottest spot-c (r = 0.572), there is a very strong positive relationship with the Average-i (r = 0.969). There is also a positive moderate relationship with the Average-c (r = 0.528) and a positive moderate relationship (r = 0.345) with the average difference.

### 3.4. The Relationship between Hottest Spot-c and Other Values

When the relationship between the highest temperature value of the contralateral joint without septic arthritis and other measurements is evaluated, it can be seen that there is a positive moderate relationship (r = 0.541) between the Hottest spot-c and the Average-i, while there is a very strong positive relationship with the Average-c (r = 0.969). A negative moderate relationship is also seen with the average difference (r = −0.540).

### 3.5. The Relationship between the Hottest Spot Difference and Other Values

When the relationship between the Hottest spot difference, the difference of the highest temperature values of the septic joint and the other non-septic joint, and other measurements is evaluated, it can be seen that there is a very strong positive relationship between the Hottest spot difference and the Coldest spot difference (r = 0.867), while there is a positive moderate relationship with the Average-i (r = 0.417). There is also a moderate negative relationship with the Average-c (r = −0.520) and a very strong positive relationship (r = 0.960) with the average difference.

## 4. Discussion

A evaluation of the temperature changes obtained by thermal camera monitoring shows that these changes could be used as a new adjunctive diagnostic tool in the differential diagnosis of septic arthritis.

### Thermal Imagers (Camera)

An infrared thermogram is an image of the temperature distribution of the captured target. Second-generation infrared detectors have not just been used for military purposes since the second half of the 20th century, but they have also been used as scanning detectors in the medical field (Jones et al., 2002) [8]. Their average speed is 1–16 frame/second, their temperature resolution is 0.5 °C, and their spatial resolution is 5 mm in a 50 cm^2^ target area (Farokhzad et al., 2020) [9]. With devices specially developed for such purposes, higher-resolution temperature distribution (better than 0.1 °C) and spatial resolution (less than 0.1 mm) could be taken at 25 frames/second (Ring et al., 2012) [10]. One of the secondary aims of this study is to lay the groundwork for the development of specially developed domestic devices suitable for medical use on this subject, which has not been studied before. In future studies, new industrial and technological projects will be produced in light of the data and outcomes obtained here. Before the 1990s, detectors had to be cooled, such as by using nitrogen or argon gas. Small-camera units developed using microbolometer detectors, which can scan superficial veins, have been equipped with features of high mobility and vertical recording (vertical mounting). This is how modern devices that do not require cooling are used today. The thermal camera used in our study is a device designed with these features (Flir^®^ brand E75 model thermal camera).

In quantitative thermal imaging, significant progress has been made in the field of infrared imaging equipment, standardization of technique, and clinical protocols within the last 20 years. The physiological mechanisms of body temperature distribution are now better understood. Further evidence has been presented regarding the accuracy of this type of thermal imaging in identifying diseases (Ring et al., 2012) [10].

Thermal imaging has great advantage regarding real time 2-dimensional temperature measurement. In modern technology, a single image contains thousands of temperature points, which can be recorded with a video recorder. The human body is homeothermic and provides the regulation and production of temperature levels necessary for survival. Relatively, the core body temperature is stable, but the tissues lining the body’s surface, particularly on the skin, are involved in the regulatory process (Jones et al., 2002) [11].

The measurement of body temperature has been carried out for generations with a simple thermometer relying on cavities, such as the oral cavity, at intervals close to a temperature of 37 °C. Thermal cameras, on the other hand, are used to detect temperature changes emerging as a result of the reflection of inflammation in an affected area to the skin temperature. The changes due to a decrease or an increase in blood flow in the clinically problematic area create this difference. Thermal imaging can be used as a medical diagnostic tool, as well as for data collection in clinical trials in principle (Ring et al., 2012) [10].

Thermal imaging, which has been investigated in terms of its utilization in many areas of orthopedics, is a means of infection detection in non-medical areas. The temperature increase due to the inflammation of plants in the field of agriculture has been demonstrated by thermal imaging methods (Farokhzad et al., 2020, Elhamahmy et al., 2016) [9,11].

In the medical field, inflammation and temperature increase caused by infection have been used in the detection of surgical wound infections and cellulitis, a skin infection (Schollemann et al., 2021, Ko et al., 2018) [12,13]. In addition, Fiz et al. (2015) found that an evaluation of the tuberculin skin test used in the diagnosis of mycobacterium tuberculosis infection by thermal imaging is significantly more effective than an evaluation by observation [14]. They also found a mean difference of 1 °C in the positive group in the area of infection (36.2 ± 1.1 °C positive group; 35.1 ± 1.6 °C negative group, *p* < 0.02, *t*-test for unpaired groups) (Fiz et al., 2015) [14].

Another important area of use for thermal imaging is diabetic foot ulcers, which is of vital importance. Van Netten et al. (2013) revealed a difference of more than 2 °C in the foot with complications in the thermal examination of the plantar surface of the ipsilateral foot and the contralateral foot of diabetic patients. They showed that the difference was more than 3 °C in patients with diffuse complications and concluded that an automated non-invasive thermogram device could be developed with these findings (van Netten et al., 2013) [15]. Based on the findings of this study, we are of the opinion that automated non-invasive devices can be developed specifically for infections, such as septic arthritis, which is the intended objective of the study. In our study, when the ipsilateral and contralateral joints were compared, it was determined that there was an average difference of 3.40 ± 1.80 °C in the septic joint (*p* < 0.000 *).

When the joints with septic arthritis and those with arthritis presentation due to other non-septic pathologies were compared, a difference of 1.21 °C was found in the septic joint (mean septic side: 37.10 ± 1.38 °C, and non-septic pathology mean: 35.89 ± 1.70 °C, *p* < 0.020 *).

As expected, the difference in inflammation caused by arthritis and inflammation caused by an infection was reflected in the difference in the temperature change detected in the joints. In the septic conditions, which require emergency surgery, the temperature of the infected joint is considerably higher than that observed in both normal joints and other arthritis cases. This finding can be considered a remarkable differential diagnostic finding. Spalding et al. (2008) evaluated the temperature distributions in active arthritis cases in the hand and wrist joints and compared 17 patients with rheumatoid arthritis and juvenile inflammatory arthritis with the control group. While the mean temperature increase was 1.0 °C ± 0.2 °C in the control group, it was 1.7 °C ± 0.6 °C in the arthritis group (*p* < 0.0001) (Spalding et al., 2008) [16]. Lasanen et al. (2015) found significantly different values in the knee joint, especially in the ankle, in the measurements they obtained from 58 children with juvenile idiopathic arthritis and arthritis caused by inflammatory diseases. In an inflamed knee, a mean increase of 1206 °C was detected, which was similar to the mean temperature increase in the non-septic arthritis group in our study (Lasanen et al., 2015) [17]. We found that the main septic arthritis cases, which were the main concern of our study, had significantly higher temperature values than the normal joints with and without arthritis (3.4 °C higher than a normal joint and 1.2 °C higher than a joint with non-septic arthritis). The pathophysiology and inflammation of septic arthritis constitute the starting point for our discussion of these findings. Apart from all other temperature-inducing joint pathologies and inflammation, the aggressive and destructive course of septic arthritis has made this situation clearer. Similar results were obtained in thermal imaging studies in which the infection foci of other body regions were evaluated, and higher values were recorded using infection-focused measurements (Schollemann et al., 2021, Ko et al., 2018) [12,13]. Zhao et al. in 2018 obtained similar results in osteomyelitis in one of the first studies in the literature on active bone lesions. In this pilot study, it was stated that thermal imaging can be used as an auxiliary diagnostic tool when compared to other diagnostic methods, such as MRI and radiography. (Zhao et al., 2018) [18]. Owen et al. prospectively examined 30 limping children admitted to an emergency department for two months in 2017, using thermal imaging. They obtained significant findings in the detection of soft tissue injuries, toddler fractures, and hip synovitis; however, a case of septic arthritis was not included in the study due to the small number of patients and the short duration of the study (Owen et al., 2017) [19]. In our study, a prospective evaluation was conducted for a year, and we collected statistically sufficient data on septic arthritis cases. Another study in the limited literature (Yusuf et al., 2015) showed the destructive effect of septic arthritis, which resulted in a higher increase in temperature compared to other arthritis, especially in patients with Gram-positive septic arthritis. In this study, 10 patients with septic arthritis out of 90 patients were detected quickly using a microcalorimetric method, based on a temperature increase at the 5th hour. The sensitivity of this temperature increase-based method was 87% with a specificity of 99% (Yusuf et al., 2015) [20]. In our literature review, the number of studies on the detection of septic arthritis with thermal cameras was found to be very limited, which can be explained by the rapid surgery of such cases when admitted to emergency services and the standardization of the thermal imaging system setup. The application of this system in an emergency room poses a challenge for this group of emergency patients. It can also be added that the incidence of septic arthritis is low compared to other arthritis, which explains the reason why there are very few studies on this patient group compared to other arthritis. Using a calorimetric analysis (differential scanning calorimetry) performed on the synovial fluids of septic arthritis cases in 2017, Dande et al. revealed that bacteria displayed higher temperatures, and even staphylococci produced a higher temperature than streptococci (Dande et al., 2017) [21]. 

In addition, a positive medium-high relationship was found in the correlation tests between the temperature, WBC, CRP, aso, leukocyte count findings in sedimentation and joint aspiration and the measurements obtained from thermal imaging, which we used for the diagnosis of septic arthritis and obtained from the patients with septic arthritis (Table 5). Thermal imaging has made great progress in the last five years in follow-up investigations of foot wounds and wound healing in diabetes, where intensive studies were carried out. These studies suggest that capillary circulation and tissue viability could easily be detected and evaluated with the most recent technological devices. 

The data obtained in this study show that thermal imaging can be effective in the differential diagnosis of septic arthritis. In the diagnosis of septic arthritis, a thermal camera can be used as a non-invasive auxiliary diagnostic tool. A temperature increase, which is an important sign of septic arthritis, can be detected by using the thermal imaging, in addition to palpation, and a quantitative value for the temperature of the infected joint can be assigned using the device. In future studies, computer-assisted devices, which are specific to the subjects, can be developed.

**Article focus**

- To use thermal imaging as a new additional non-invasive diagnostic tool in the differential diagnosis of septic arthritis.
- To compare the temperature increase and changes between septic and non-septic arthritis.
- To assign a quantitative value for the local temperature increase in the infected joint, in addition to palpation.

**Key messages**

- In the diagnosis of septic arthritis, a high-resolution thermal imager can be used as an auxiliary non-invasive diagnostic tool.
- A temperature increase, which is an important sign of septic arthritis, can be detected by using the thermal imaging, in addition to palpation.
- Specially designed thermal devices with special software for septic arthritis can be developed.

**Strengths and limitations**

- Although the imaging in this study was performed with a high-resolution and sensitive thermal camera, there is a minimal level of uncertainty regarding its accuracy due to internal and external factors, such as humidity. Further research should be directed at whether skin surface temperature differences can be used to differentiate between various mimicking clinical diseases.
- This cohort study’s findings are not sufficient to exclude the necessity of an invasive intra-articular aspiration. More comprehensive research studies are needed.

## Figures and Tables

**Table 1:** Statistics of categorical variables.

		*n*	%
Gender	Male	27	55.1
	Woman	22	44.9
Localization	Right knee	26	53.1
	Left knee	23	46.9
Reproduction in the joint	No	34	69.4
	Yes	15	30.6
Septic arthritis	No	34	69.4
	yes	15	30.6

**Table 2:** Descriptive statistics of measurements.

	Minimum	Maximum	Mean	ss
Temperature	36.00	39.50	37.02	0.94
Wbc	5.19	20.99	9.49	4.30
Aso	51.90	720.00	253.49	136.33
Crp	0.10	293.00	20.01	50.03
Sedimentation	1.00	168.00	73.57	41.41
Leukocyte count in joint fluid (mm^3^)	800.00	120,000.00	38,463.27	35,764.33
Coldest spot	29.20	38.40	34.64	2.19
Hottest spot-i	33.50	40.60	37.14	1.68
Coldest spot-c	28.70	37.90	33.00	2.10
Hottest spot-c	31.60	39.60	35.50	1.77
Hottest spot difference	−1.20	7.00	1.64	1.60
Coldest spot difference	−2.70	7.10	1.64	2.11
Average-i	32.80	39.50	36.26	1.69
Average-c	30.30	38.50	34.57	1.93
Average difference	−1.40	7.30	1.69	1.78

**Table 3:** Evaluation of the measurements in terms of septic arthritis.

	Septic Arthritis				Test Statistics	*p*
	No		Yes			
	Mean	ss	Mean	ss		
Temperature	36.98	0.89	37.12	1.07	−0.467	0.642
Wbc	9.22	3.97	10.09	5.09	−0.650	0.519
Aso	208.47	105.13	355.53	146.93	−3.983	0.000
^a^ Crp	18.33	53.03	23.81	43.96	165.000	0.051
Sedimentation	62.24	37.83	99.27	38.58	−3.140	0.003
Leukocyte count in joint fluid mm^3^	22,026.47	22,317.71	75,720.00	32,681.21	−6.703	0.000
Coldest spot	34.14	2.24	35.77	1.62	−2.533	0.015
Hottest spot-i	36.79	1.71	37.93	1.34	−2.272	0.028
Coldest spot-c	33.36	1.71	32.19	2.68	1.557	0.136
Hottest spot-c	35.82	1.51	34.79	2.15	1.917	0.061
Hottest spot difference	0.98	1.03	3.13	1.67	−5.540	0.000
Coldest spot difference	0.78	1.60	3.58	1.84	−5.398	0.000
Average-i	35.89	1.70	37.10	1.38	−2.417	0.020
Average-c	34.95	1.60	33.70	2.38	1.864	0.077
Average difference	0.94	1.16	3.40	1.80	−4.867	0.000

a = Mann–Whitney test. The others are independent-group *t*-tests.

**Table 4:** The test results presenting the differences in the measurements by age and septic arthritis status.

Septic Arthritis		Age				Test Statistics	*p*
		Under 18		Over 18			
		Mean	ss	Mean	ss		
No	Temperature	36.72	0.79	37.15	0.93	−1.389	0.174
	Wbc	10.11	4.22	8.67	3.80	1.024	0.313
	Aso	219.45	128.36	201.67	90.68	0.474	0.639
	^a^ Crp	35.30	84.69	7.82	5.76	96.000	0.151
	Sedimentation	64.38	43.67	60.90	34.81	0.257	0.799
	Leukocyte count in joint fluid mm^3^	21,592.31	17,803.30	22,295.24	25,128.86	−0.088	0.931
	Coldest spot-i	33.12	2.41	34.78	1.92	−2.230	0.033
	Hottest spot-i	36.53	1.48	36.96	1.86	−0.701	0.489
	Coldest spot-c	33.02	1.59	33.57	1.78	−0.906	0.371
	Hottest spot-c	36.11	1.15	35.64	1.69	0.881	0.385
	Hottest spot difference	0.42	0.65	1.32	1.09	−2.677	0.012
	Coldest spot difference	0.09	1.44	1.21	1.57	−2.079	0.046
	Average-i	35.67	1.49	36.03	1.83	−0.602	0.551
	Average-c	35.22	1.32	34.79	1.75	0.772	0.446
	Average difference	0.45	0.75	1.25	1.27	−2.059	0.048
Yes	Temperature	37.26	1.18	37.05	1.07	0.347	0.734
	Wbc	10.53	7.45	9.88	3.93	0.225	0.825
	Aso	347.80	216.21	359.40	112.95	−0.139	0.892
	^a^ Crp	10.82	10.77	30.31	53.05	18.000	0.391
	Sedimentation	71.60	31.91	113.10	34.99	−2.224	0.045
	Leukocyte count in joint fluid mm^3^	55,560.00	44,088.18	85,800.00	21,420.65	−1.451	0.207
	Coldest spot-i	36.74	1.42	35.29	1.55	1.749	0.104
	Hottest spot-i	38.52	1.64	37.63	1.14	1.239	0.237
	Coldest spot-c	33.10	3.22	31.74	2.42	0.923	0.373
	Hottest spot-c	35.56	2.68	34.41	1.88	0.973	0.348
	Hottest spot difference	2.96	2.49	3.22	1.24	−0.275	0.788
	Coldest spot difference	3.64	2.62	3.55	1.48	0.086	0.933
	Average-i	37.82	1.58	36.74	1.20	1.489	0.160
	Average-c	34.64	2.63	33.23	2.23	1.090	0.296
	Average difference	3.18	2.44	3.51	1.53	−0.324	0.751

a = Mann–Whitney Test. The others are independent-group *t*-tests.

**Table 5:** The results of the correlation test performed to examine the relationship between the measurements.

	Temp.	Wbc	Aso	Crp	Sedimentation	Leukocyte Count in Joint Fluid mm^3^	Coldest Spot-i	Hottest Spot-i	Coldest Spot-c	Hottest Spot-c	Hottest Spot Difference	Coldest Spot Difference	Average-i	Average-c	Average Difference
Temperature	1	0.116	0.295	0.022	0.040	0.018	0.338	0.252	0.237	0.117	0.135	0.115	0.243	0.116	0.105
Wbc		1	−0.095	0.181	0.043	0.121	−0.073	−0.055	−0.172	−0.275	0.247	0.095	−0.114	−0.320	0.240
Aso			1	0.492	0.420	0.424	0.367	0.419	0.104	0.144	0.280	0.278	0.419	0.092	0.297
Crp				1.000	0.418	0.225	0.427	0.325	0.126	−0.061	0.410	0.367	0.305	−0.034	0.376
Sedimentation					1	0.491	0.284	0.284	−0.065	−0.071	0.377	0.360	0.274	−0.090	0.358
Leukocyte count in joint fluid mm^3^						1	0.198	0.171	−0.361	−0.367	0.587	0.565	0.219	−0.346	0.583
Coldest spot-i							1	0.796	0.518	0.380	0.414	0.524	0.840	0.396	0.366
Hottest spot-i								1	0.438	0.572	0.416	0.391	0.969	0.528	0.345
Coldest spot-c									1	0.811	−0.439	−0.457	0.426	0.855	−0.524
Hottest spot-c										1	−0.509	−0.412	0.541	0.969	−0.540
Hottest spot difference											1	0.867	0.417	−0.520	0.960
Coldest spot difference												1	0.449	−0.439	0.902
Average-i													1	0.523	0.380
Average-c														1	−0.590
Average difference															1

## Data Availability

The data presented in this study are available on request from the corresponding author. The data are not publicly available due to privacy reasons.
